# Association of air pollution exposure with exercise-induced oxygen desaturation in COPD

**DOI:** 10.1186/s12931-022-02000-1

**Published:** 2022-03-31

**Authors:** Kang-Yun Lee, Sheng-Ming Wu, Hsiao-Yun Kou, Kuan-Yuan Chen, Hsiao-Chi Chuang, Po-Hao Feng, Kian Fan Chung, Kazuhiro Ito, Tzu-Tao Chen, Wei-Lun Sun, Wen-Te Liu, Chien-Hua Tseng, Shu-Chuan Ho

**Affiliations:** 1 Division of Pulmonary Medicine, Department of Internal Medicine, Shuang Ho Hospital, Taipei Medical University, New Taipei City, Taiwan; 2 Division of Pulmonary Medicine, Department of Internal Medicine, School of Medicine, College of Medicine, Taipei Medical University, Taipei, Taiwan; 3 School of Respiratory Therapy, College of Medicine, Taipei Medical University, Taipei, Taiwan; 4 Cell Physiology and Molecular Image Research Center, Taipei Municipal Wan Fang Hospital, Taipei, Taiwan; 5 National Heart and Lung Institute, Imperial College London, London, UK

**Keywords:** COPD, Emphysema, Exercise-induced desaturation (EID), Air pollution, Low attenuation area (LAA), Dynamic hyperinflation (DH)

## Abstract

**Background:**

There is a link between exposure to air pollution and the increased prevalence of chronic obstructive pulmonary disease (COPD) and declining pulmonary function, but the association with O_2_ desaturation during exercise in COPD patients with emphysema is unclear. Our aims were to estimate the prevalence of O_2_ desaturation during exercise in patients with COPD, and determine the association of exposure to air pollution with exercise-induced desaturation (EID), the degree of emphysema, and dynamic hyperinflation (DH).

**Methods:**

We assessed the effects of 10-year prior to the HRCT assessment and 7 days prior to the six-minute walking test exposure to particulate matter with an aerodynamic diameter of < 10 µm (PM_10_) or of < 2.5 µM (PM_2.5)_, nitrogen dioxide (NO_2_), and ozone (O_3_) in patients with emphysema in this retrospective cohort study. EID was defined as a nadir standard pulse oximetry (SpO_2_) level of < 90% or a delta (△)SpO_2_ level of ≥ 4%. Ambient air pollutant (PM_2.5_, PM_10_, O_3_, and NO_2_) data were obtained from Taiwan Environmental Protection Administration (EPA) air-monitoring stations, usually within 10 km to each participant’s home address.

**Results:**

We recruited 141 subjects with emphysema. 41.1% of patients with emphysema exhibited EID, and patients with EID had more dyspnea, worse lung function, more severe emphysema, more frequent acute exacerbations, managed a shorter walking distance, had DH, and greater long-term exposure to air pollution than those without EID. We observed that levels of 10-year concentrations of PM_10_, PM_2.5_, and NO_2_ were significantly associated with EID, PM_10_ and PM_2.5_ were associated with the severity of emphysema, and associated with DH in patients with emphysema. In contrast, short-term exposure did not have any effect on patients.

**Conclusion:**

Long-term exposure to ambient PM_10_, PM_2.5_ and NO_2_, but not O_3_, was associated with EID.

**Supplementary Information:**

The online version contains supplementary material available at 10.1186/s12931-022-02000-1.

## Background

Chronic Obstructive Pulmonary Disease (COPD) is the third leading cause of death globally, it is a preventable and treatable lung disease[1]. COPD is considered a chronic inflammatory process throughout the airways and lung parenchyma, that is characterized by progressive development of limited airflow[2] destruction of lung parenchyma (emphysema), and sputum production [3]. Emphysema is associated with increasing dyspnea due to destruction of the alveolar wall, with a consequent reduction in the surface area available for oxygen exchange. In turn, the oxygen (O_2_) level in the bloodstream is reduced, and impaired O_2_ transport and utilization are common consequences of pulmonary emphysema, which lead to ventilation/perfusion (V/Q) mismatch resulting in O_2_ desaturation during exercise.

Exercise-induced desaturation (EID) is defined as the nadir of standard pulse oximetry (SpO_2_) level of < 90% or a reduction in (△)SpO_2_ level of ≥ 4% [4, 5]. The 6-min walking test (6MWT) is a standard O_2_ desaturation test that uses a pulse oximeter for continuous measurement to identify EID [6–8]. A previous study found that EID in COPD patients was related to reduced exercise performance, severe airflow limitations, V/Q mismatch, diffusion limitation, muscle weakness, impaired daily physical activity, and dynamic hyperinflation (DH). DH is a pathophysiologic consequence of airflow limitation during exercise in patients with COPD and an important contributing factor to dyspnea[9]. Delta (Δ) inspiratory capacity (IC) of ≤ 0.100 L at the peak of exercise is considered to define patients with DH [10, 11].

The human health effects of exposure to air pollutants, which include both particulate matter (PM) and gaseous contaminants, have gained prominence as a global public health concern. Because the respiratory tract is the primary portal of entry for air pollutants, the respiratory effects of pollutants have been studied for decades [12]. Chronic exposure to noxious gases, smoking, and air pollution are major risk factors for COPD. Ambient air pollution has adverse effects on lung function in COPD patients [13], especially for long-term exposure which is associated with increasing risk of developing emphysema [14]. Previous studies have shown that short-term events of air pollution may affect lung function decrement and even the impairment in lung[15]. Some studies reported that EID occurs in 20% ~ 50% of patients with COPD, but the association between EID and air pollution has not been clarified, especially with long-term or short-term exposure. We hypothesized that air pollution is a factor causing O_2_ desaturation during exercise in patients with emphysema. Thus, the aims of this study were to estimate the prevalence of O_2_ desaturation during exercise in a cohort population of patients with emphysematous COPD, and to determine whether air pollution was associated with EID, the severity of emphysema, and DH.

## Materials and methods

### Study subjects

This retrospective cross-sectional study was conducted between January 2017 and December 2020 in a hospital in New Taipei City, Taiwan. In total, 141 participants were recruited from a COPD cohort of the Respiratory Department. The inclusion criteria were (1) aged 40 ~ 85 years; (2) having been diagnosed with COPD, defined as having a post-bronchodilator forced expiratory volume in the first second (FEV1)/forced vital capacity (FVC) ratio of ≤ 70%; (3) having undergone chest high-resolution computed tomography (HRCT); (4) having undergone a 6-min walking test (6MWT) and being less than 6 months from the date of a pulmonary function test; (5) having a stable condition and no acute exacerbations (AE) within 3 months, defined as no requirement for antibiotics or oral corticosteroid treatment, and no change in respiratory symptoms; and (6) air pollution data available from nearby monitoring station. The exclusion criteria were (1) AE during 3 months prior to the study or (2) having a mental disability such that the patient was unable to complete the 6MWT. The study protocol was approved by the Ethics Committee of Taipei Medical University (approval no. N201902008).

Demographic and lifestyle data of participants were collected from hospital medical records, including sex, body-mass index (BMI), smoking pack-years, and number of AEs in the past year[16]. AE was determined by an increase in respiratory symptoms (including cough, dyspnea, and sputum production) and needing additional therapy such as corticosteroids. A patient going to the emergency room or being admitted because of respiratory symptoms was also included in the AE counts. The modified Medical Research Council (mMRC) is a simple commonly used instrument to characterize the impacts of dyspnea, disability, and functioning on daily activities; the impact of the disease was assessed with the previously validated Chinese version of the COPD assessment test (CAT) for conducting dyspnea assessments.

### High-resolution computed tomography (HRCT)

HRCT scans were acquired at suspended full inspiration. GE Discovery CT 750 HD (GE, Fort Myers, Florida, USA) was performed with 10-mm slice thicknesses. Emphysema was defined by the percent of voxels with Hounsfield units (HU) of < − 950 (% low attenuation area, LAA) on CT[17, 18]. The LAA is an area where the density on a CT scan is below a fixed threshold, related to the total lung area (LAA%) [19]. LAA data were analyzed by professional radiologists. The severity of emphysema was classified as none (LAA < 5%), mild (LAA 5–10%), moderate (LAA 10–20%), or severe (LAA ≥ 20%)[20].

### Air pollution analysis

Air pollutant (PM_2.5_, PM_10_, O_3_, and NO_2_) data were obtained from Taiwan Environmental Protection Administration (EPA) air-monitoring stations (https://airtw.epa.gov.tw/). Ambient Individual-level exposure to single air pollutants were predicted by a hybrid kriging/land-use regression (hybrid kriging-LUR) approach, which was previously demonstrated[21–25]). Daily air pollution exposure data were assigned to individuals on the basis of the participant’s home address. The daily average concentrations of air pollution data in the preceding years were computed for subsequent analyses. The nearest 3 air monitoring stations were identified using the ArcGIS server software (ESRI, Redlands, CA, USA), and then air pollution data was extracted. The distance range between the nearest 3 stations and the participants’ home addresses was an average of 2.35–4.73 km. The vehicle emissions in the greater Taipei area contributed to > 90% of CO, 80% of NO_X_, and nearly 50% of PM_2.5_ in the downtown areas of Taipei [26]. Taking into account that the point data of the monitoring points are not consistent with the actual surface, the air has a strong diffusion effect, and the inverse distance weighted interpolation method is used to estimate the daily average concentration of air pollution data. [27]. Traffic emissions are the source of approximately 50% of the PM2.5 in the greater Taipei area[26]. Average individual exposure to air pollution were estimated for 10 years prior to the HRCT assessment and 7 days prior to the six-minute walking test.

### Pulmonary function and 6-min walking test (6MWT)

A pulmonary function test was conducted using a Vitalograph Spirotac V™ (Vitalograph, MK18 1SW, UK) after a 10-min rest; the post-bronchodilator FEV1 and FVC were measured, and the FEV1/FVC ratio was calculated[28]. The 6MWT was conducted in all patients, who were instructed to walk as far as possible but were allowed to rest and stop during the test according to American Thoracic Society (ATS) guidelines [29]. Oxygen saturation was recorded using a continuous finger-adapted pulse oximeter during the 6MWT. The inspiratory capacity (IC) was measured before and immediately after the 6MWT. All participants were familiar with the 6MWT before the test.

### Statistical analysis

Descriptive statistics were recorded for each variable of the characteristics of subjects and exercise-induced desaturation, with quantitative data shown as the mean and standard deviation (SD). Differences in comparisons between two groups were analyzed with an independent *t*-test and Mann–Whitney *U*-test. A logistic regression analysis was performed to determine air pollution factors associated with EID, emphysema severity, %LAA, and DH. To evaluate the impact of air pollution on EID, with the no EID group as a reference, the crude odds ratio (OR) was calculated through a univariate logistic regression model. Adjusted OR were calculated for specific EID, with no EID group as the reference, adjusting for age, sex, and smoking pack-years by the binary logistic regression. The level of significance was set to α = 0.05. Data were analyzed with IBM SPSS Statistic 20 for Windows (IBM, SPSS, Chicago, IL, USA) and GraphPad Prism 7 (GraphPad Software, La Jolla, CA, USA).

## Results

### Characteristics of study participants

**Fig. 1:**
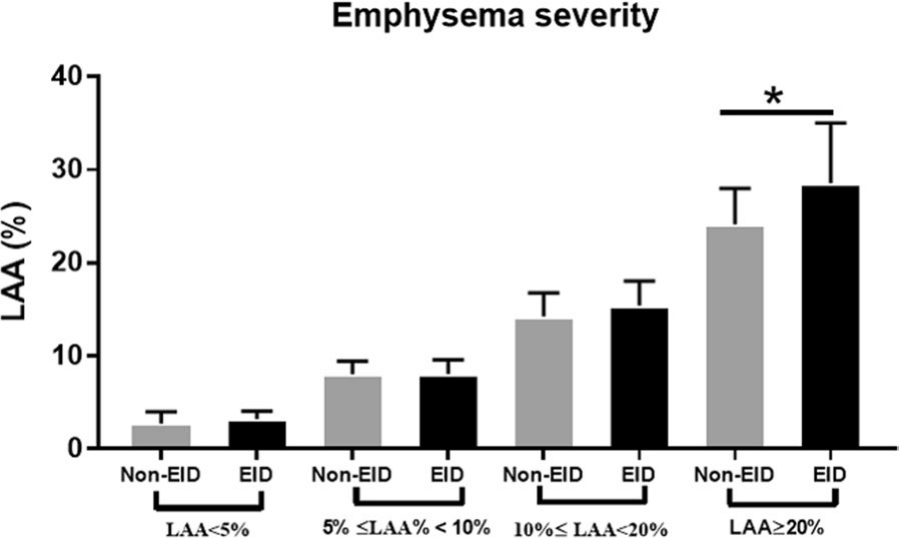
Comparison of the severity of emphysema low attenuation area (LAA) % in non-exercise-induced desaturation (EID) vs. EID groups

Patient characteristics are shown in Table 1. Eighty-three patients (77 men and 6 women, with a mean age of 68.65 ± 8.16 years) in the non-EID group and 58 patients (49 men and 9 women, with a mean age of 70.31 ± 7.51 years) in the EID group were enrolled in this study. Most patients without or with EID had a history of smoking (91.5%;46.52 ± 33.56 vs.54.00 ± 37.36 smoking pack-years, respectively, *p* > 0.05). The mMRC dyspnea sensation score, severe emphysema (Fig. 1), and AE were significantly higher in patients with EID than in those without EID (all *p* < 0.01). EID patients had significantly lower values for the 6MWD and ΔIC than did non-EID patients (*p* < 0.05). No significant differences were observed in age, sex, BMI, smoking pack-years, or CAT between the two groups (*p* > 0.05).

**Table 1:** Demographic characteristics, clinical, and air pollution variables in non-exercise-induced desaturation (EID) and EID groups (*N* = 141)

**Variable**	All patients (*N* = 141)	Non-EID (*N* = 83)	EID (*N* = 58)	*p* value
Age (years)	69.33 ± 7.91	68.65 ± 8.156	70.31 ± 7.51	0.2216
Sex (M/F)	126/15	77/6	49/9	0.3981
BMI (kg/m^2^)	23.25 ± 4.11	23.68 ± 4.01	22.62 ± 4.21	0.1341
Smoking status				0.0012
Smoker (n, %)	62 (44.0)	46 (55.42)	16 (27.59)	
Ex-smoker (n, %)	67 (47.5)	32 (38.55)	35 (60.34)	
Never smoking (n, %)	12 (8.5)	5 (6.02)	7 (12.07)	
Smoking (pack-years)	49.60 ± 35.24	46.52 ± 33.56	54.00 ± 37.36	0.2159
mMRC	1.33 ± 1.07	1.04 ± 0.92	1.76 ± 1.13	< 0.0001
CAT	9.54 ± 6.98	8.93 ± 7.12	10.4 ± 6.75	0.2218
Emphysema severity	15.77 ± 9.44	12.50 ± 6.96	20.45 ± 10.55	< 0.0001
None (*n*, %)	28 (19.86)	14 (16.87)	4 (6.90)	
Mild (*n*, %)	26 (18.44)	18 (21.69)	8 (13.79)	
Moderate (*n*, %)	54 (38.30)	38 (45.78)	16 (27.59)	
Severe (*n*, %)	43 (30.50)	13 (15.66)	30 (51.72)	
AE (time/year)	0.81 ± 1.53	0.43 ± 0.65	1.35 ± 2.13	0.0008
Pulmonary function test				
FEV1 (pred %)	55.38 ± 19.96	62.45 ± 17.76	43.87 ± 18.36	< 0.0001
FVC (pred %)	78.73 ± 19.74	83.09 ± 18.42	71.01 ± 19.34	0.0003
6 min walking test				
(6MWD) (m)	375.1 ± 114.1	398.5 ± 101.0	341.8 ± 124.0	0.0034
SpO_2_-pre (%)	94.11 ± 2.34	94.93 ± 1.89	92.95 ± 2.44	< 0.0001
SpO_2_-post (%)	89.06 ± 5.48	92.78 ± 1.84	83.72 ± 4.45	< 0.0001
ΔIC (L)	-0.05 ± 0.26	0.01 ± 0.23	-0.14 ± 0.29	0.0025
Air pollution				
PM_10_ (μg/m^3^)-7 day	32.13 ± 10.62	32.12 ± 10.97	32.15 ± 10.13	0.9894
PM_2.5_ (μg/m^3^)-7 day	16.30 ± 5.44	15.98 ± 5.20	16.82 ± 5.82	0.3954
NO_2_ (ppb)-7 day	17.59 ± 3.79	17.74 ± 3.75	17.34 ± 3.89	0.5545
O_3_ (ppb)-7 day	29.43 ± 6.55	28.56 ± 5.89	30.86 ± 7.35	0.0526
PM_10_ (μg/m^3^)-10 year	40.71 ± 2.51	40.19 ± 2.35	41.57 ± 2.54	0.0021
PM_2.5_ (μg/m^3^)-10 year	22.64 ± 1.70	22.26 ± 1.49	23.24 ± 1.85	0.0012
NO_2_ (ppb)-10 year	20.30 ± 1.84	19.85 ± 1.76	20.88 ± 1.76	0.0007
O_3_ (ppb)-10 year	26.18 ± 0.98	26.24 ± 1.02	26.07 ± 0.90	0.3398
Exposure season^#^				0.9801
Spring (n, %)	39 (27.66)	25 (30.12)	14 (24.14)	
Summer (n, %)	30 (21.28)	17 (20.48)	13 (22.41)	
Fall (n, %)	46 (32.62)	25 (30.12)	21 (36.21)	
Winter (n, %)	26 (18.44)	16 (19.28)	10 (17.24)	

Data are presented as % or mean ± standard deviation. Severity of emphysema was classified as none (low attenuation area (LAA) < 5%), mild (LAA 5–10%), moderate (LAA 10–20%), or severe (LAA ≥ 20%); *SpO*_*2*_ oxyhemoglobin saturation by pulse oximetry, *ΔSpO*_*2*_* (%)* post-exercise saturation—pre-exercise saturation, *ΔIC* post-exercise inspiratory capacity—pre-exercise inspiratory capacity, *BMI* body-mass index, *AE* acute exacerbation, *LAA* low attenuation area, *CAT* chronic obstructive pulmonary disease assessment test, *IC* inspiratory capacity, *pred* predicted, *6MWD* 6 min walking distance, *PM*_*10*_ particulate matter of < 10 μm in aerodynamic diameter, *PM*_*2.5*_ particulate matter of < 2.5 μm in aerodynamic diameter, *NO*_*2*_ nitrogen dioxide, *O*_*3*_ ozone. ^#^Exposure season:Spring (March–May), Summer (June–August), Fall (September–November), Winter (December–February)

Levels of 10-year concentrations of PM_10_ (mean 41.57 ± 2.54 vs. 40.19 ± 2.35 μg/m^3^), PM_2.5_ (mean 23.24 ± 1.85 vs. 22.26 ± 1.49 μg/m^3^), and NO_2_ (mean 20.88 ± 1.76 vs. 19.85 ± 1.76 ppb) were significantly higher in patients with EID than in those without EID (all *p* < 0.01). No significant difference was observed in O_3_ levels between the two groups (*p* > 0.05). Short-term exposure did not show any effect on patients. Levels of 7 days concentrations of air pollution matters (PM_10_, PM_2.5_, NO_2_, and O_3_) and exposure season had no significant differences in patients with EID and without EID (all p > 0.05).

### Associations of air pollution with exercise-induced desaturation (EID)

**Fig. 2:**
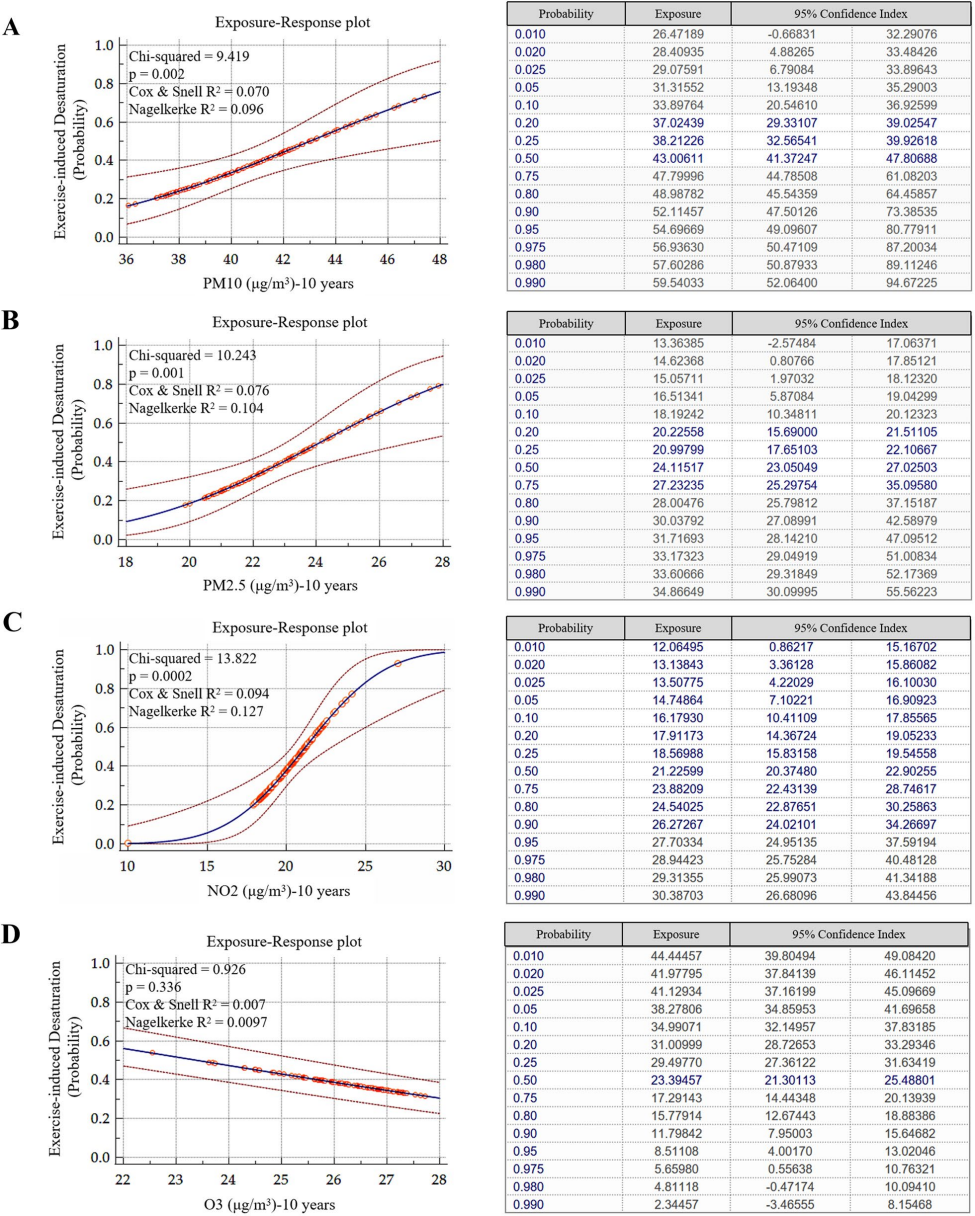
Exposure–response plots (*left*) and charts (*right*) showing the probability of exercise-induced desaturation in our study cohort (n = 141) upon exposure to varying concentration of **A** PM_10_, **B** PM_2.5_, **C** NO_2_, or **D** O_3_ over a period of 10 years. *PM*_*10*_ particulate matter of < 10 μm in aerodynamic diameter, *PM*_*2.5*_ particulate matter of < 2.5 μm in aerodynamic diameter, *NO*_*2*_ nitrogen dioxide, *O*_*3*_ ozone

Results of the logistic regression and sensitivity analysis are presented in Table 2a, b with air pollution and exercise-induced oxygen desaturation as dependent variables. We observed that a 1-μg/m^3^ increase in PM_10_ was associated with a 1.258-fold increase in the crude odds ratio (OR) of EID (95% confidence interval (CI): 1.080 ~ 1.466; *p* < 0.01). After adjusting for age, sex, and smoking pack-year, a 1-μg/m^3^ increase in PM_10_ was associated with a 1.288-fold increase in the adjusted OR of EID (95% CI: 1.070 ~ 1.492; *p* < 0.01); after adjusting for age, sex, smoking pack-year and seasonal effects, a 1-μg/m^3^ increase in PM_10_ was associated with a 1.306-fold increase in the adjusted OR of EID (95% CI: 1.108 ~ 1.539; *p* < 0.01); after adjusting for age, sex, smoking pack-year, seasonal effects and AE frequency, a 1-μg/m^3^ increase in PM_10_ was associated with a 1.313-fold increase in the adjusted OR of EID (95% CI: 1.096 ~ 1.574; *p* < 0.01). Consistent with these data, our exposure–response analysis showed that the probability of EID upon exposure to PM_10_ over time was concentration-dependent and statistically significant (*X*^2^ = 9.419, *p* = 0.002, Cox & Snell R^2^ = 0.070, Nagelkerke R^2^ = 0.096) (Fig. 2A). Next, we found a strong positive correlation between PM_2.5_ exposure and EID (*X*^2^ = 10.243, *p* = 0.001, Cox & Snell R^2^ = 0.076, Nagelkerke R^2^ = 0.104) (Fig. 2B), and as shown in Table 2, 1 μg/m^3^ increase in PM_2.5_ was associated with a 1.424-fold increase in the crude OR of EID (95% CI: 1.135–1.787; *p* < 0.01). After adjusting for age, sex, and smoking pack-year, a 1-μg/m^3^ increase in PM_2.5_ was associated with a 1.476-fold increase in the adjusted OR of EID (95% CI: 1.166–1.869; *p* < 0.01); after adjusting for age, sex, smoking pack-year and seasonal effects, a 1-μg/m^3^ increase in PM_2.5_ was associated with a 1.502-fold increase in the crude OR of EID (95% CI: 1.178–1.917; *p* < 0.01); after adjusting for age, sex, smoking pack-year, seasonal effects and AE frequency, a 1-μg/m^3^ increase in PM_2.5_ was associated with a 1.471-fold increase in the adjusted OR of EID (95% CI: 1.138–1.903; *p* < 0.01). A 1-ppb increase in NO_2_ was associated with a 1.514-fold increase in the crude OR of EID (95% CI: 1.184–1.934; *p* < 0.01). After adjusting for age, sex, and smoking pack-year, a 1-ppb increase in NO_2_ was associated with a 1.518-fold increase in the adjusted OR of EID (95% CI: 1.172–1.965; *p* < 0.01); after adjusting for age, sex, smoking pack-year and seasonal effects, a 1-ppb increase in NO_2_ was associated with a 1.654-fold increase in the adjusted OR of EID (95% CI: 1.257–2.177; *p* < 0.01); after adjusting for age, sex, smoking pack-year, seasonal effects and AE frequency, a 1-ppb increase in NO_2_ was associated with a 1.800-fold increase in the adjusted OR of EID (95% CI: 1.308–2.476; *p* < 0.01). In line with these data, we also observed a very significant positive correlation between patients’ exposure to NO_2_ and EID (*X*^2^ = 13.822, *p* = 0.002, Cox & Snell R^2^ = 0.094, Nagelkerke R^2^ = 0.127) (Fig. 2C). On the contrary, we found that increased exposure to O3 was associated with decreased probability of EID (*X*^2^ = 0.926, *p* = 0.336, Cox & Snell R^2^ = 0.007, Nagelkerke R^2^ = 0.01) (Fig. 2D). The logistic regression and sensitivity analysis predicting an EID in short-term air pollution exposure did not show significant difference (Additional file 1: Table S2a and S2b).

**Table 2:** **a** Logistic regression predicting an exercise-induced desaturation of ≥ 4% and SpO2 of < 90% during the 6-min walking test. **b** Sensitivity analysis predicting an exercise-induced desaturation of ≥ 4% and SpO2 of < 90% during the 6 min walking test

a				
Variable	Crude OR (95% CI)	*p* value	Adjusted OR (95% CI)*	*p* value
PM_10_(μg/m^3^)	1.258 (1.080–1.466)	0.003	1.288 (1.099–1.509)	0.002
PM_2.5_(μg/m^3^)	1.424 (1.135–1.787)	0.002	1.476 (1.166–1.869)	0.001
NO_2_(ppb)	1.514(1.184–1.935)	0.001	1.584 (1.223–2.053)	< 0.001
O_3_(ppb)	0.838 (0.583–1.204)	0.339	0.812 (0.558–1.182)	0.276

b				
Variables	Adjust OR^#^ (95%CI)	P value	Adjust OR^¥^ (95%CI)	P value
PM_10_(μg/m^3^)	1.306 (1.108–1.539)	0.001	1.313 (1.096–1.574)	0.003
PM_2.5_(μg/m^3^)	1.502 (1.178–1.917)	0.001	1.471 (1.138–1.903)	0.003
NO_2_(ppb)	1.654 (1.257–2.177)	< 0.001	1.800 (1.308–2.476)	< 0.001
O_3_(ppb)	0.812 (0.554–1.189)	0.283	0.664 (0.405–1.090)	0.105

*PM*_*10*_ particulate matter of < 10 μm in aerodynamic diameter, *PM*_*2.5*_ particulate matter of < 2.5 μm in aerodynamic diameter, *NO*_*2*_ nitrogen dioxide, *O*_*3*_ ozone, *OR* odds ratio, *CI* confidence interval

*Multivariable logistic regression adjusted for age, sex, smoking pack-year

^**#**^Multivariable logistic regression adjusted for age, sex, smoking pack-year and seasonal effects

^**¥**^Multivariable logistic regression adjusted for age, sex, smoking pack-year, seasonal effects and AE frequency

### Associations of PM_10_ and PM_2.5_ with emphysema severity

We also did a subgroup analysis by emphysema severity and DH to eliminate potential effects. Associations of air pollution with emphysema severity and dynamic hyperinflation are shown in Table 3a, b. We observed that a 1-μg/m^3^ increase in PM_2.5_ was associated with a 1.255-fold increase in the crude OR of severe emphysema LAA of ≥ 20% (95% CI: 1.001–1.573; *p* < 0.05). After adjusting for age, sex, and smoking pack-years, a 1-μg/m^3^ increase in PM_2.5_ was associated with a 1.273-fold increase in the adjusted OR of severe emphysema LAA of ≥ 20% (95% CI: 1.006–1.612; *p* < 0.05);after adjusting for age, sex, smoking pack-year and seasonal effects, a 1-μg/m^3^ increase in PM_2.5_ was associated with a 1.304-fold increase in the crude OR of severe emphysema LAA of ≥ 20% (95% CI: 1.022–1.663; *p* < 0.05); after adjusting for age, sex, smoking pack-year, seasonal effects and AE frequency, a 1-μg/m^3^ increase in PM_2.5_ was associated with a 1.343-fold increase in the adjusted OR of severe emphysema LAA of ≥ 20% (95% CI: 1.029–1.752; *p* < 0.05). We observed that a 1-μg/m3 increase in PM_10_ was associated with a 1.137-fold increase in the crude OR of severe emphysema LAA of ≥ 20% (95% CI: 0.972–1.329; p > 0.05). After adjusting for age, sex, smoking pack-year, seasonal effects and AE frequency, a 1-μg/m^3^ increase in PM_10_ was associated with a 1.219-fold increase in the adjusted OR of severe emphysema LAA of ≥ 20% (95% CI: 1.007–1.476; *p* < 0.05). However, the logistic regression and sensitivity analysis predicting LAA in severe emphysema of patients with short-term air pollution exposure did not show significant difference (Additional file 1: Table S3a and S3b).

**Table 3:** **a** Logistic regression predicting severe emphysema low attenuation area (LAA) of ≥ 20%. **b** Sensitivity analysis predicting severe emphysema low attenuation area (LAA) of ≥ 20%

a				
Variable	Crude OR (95% CI)	*p* value	Adjusted OR (95% CI)*	*p* value
PM_10_(μg/m^3^)	1.137 (0.972–1.329)	0.108	1.151 (0.978–1.355)	0.091
PM_2.5_(μg/m^3^)	1.255 (1.001–1.573)	0.049	1.273 (1.006–1.612)	0.044
NO_2_(ppb)	1.086 (0.911–1.295)	0.359	1.100 (0.908–1.333)	0.332
O_3_(ppb)	0.919 (0.620–1.363)	0.676	0.906 (0.605–1.357)	0.633

b				
Variables	Adjust OR^#^ (95%CI)	P value	Adjust OR^¥^ (95%CI)	P value
PM_10_(μg/m^3^)	1.174 (0.991–1.390)	0.063	1.219 (1.007–1.476)	0.042
PM_2.5_(μg/m^3^)	1.304 (1.022–1.663)	0.032	1.343 (1.029–1.752)	0.030
NO_2_(ppb)	1.069 (0.853–1.340)	0.562	1.158 (0.873–1.537)	0.309
O_3_(ppb)	0.899 (0.594–1.363)	0.617	0.704 (0.419–1.186)	0.187

PM_10_, particulate matter of <10 μm in aerodynamic diameter; PM_2.5_, particulate matter of < 2.5 μm in aerodynamic diameter; NO_2_, nitrogen dioxide; O_3_, ozone; OR, odds ratio; CI, confidence interval

*Multivariable logistic regression adjusted for age, sex, smoking pack-year

^**#**^Multivariable logistic regression adjusted for age, sex, smoking pack-year and seasonal effects

^**¥**^Multivariable logistic regression adjusted for age, sex, smoking pack-year, seasonal effects and AE frequency

### Associations of PM_10_ and PM_2.5_ with dynamic hyperinflation (DH)

In Table 4a, b, one can observe that a 1-μg/m^3^ increase in PM_10_ was associated with a 1.222-fold increase in the crude OR of DH (95% CI: 1.021–1.463; *p* < 0.05). After adjusting for age, sex, and smoking pack-years, a 1-μg/m^3^ increase in PM_10_ was associated with a 1.269-fold increase in the adjusted OR of DH (95% CI: 1.046–1.540; *p* < 0.05); after adjusting for age, sex, smoking pack-year and seasonal effects, a 1-μg/m^3^ increase in PM_10_ was associated with a 1.281-fold increase in the adjusted OR of DH (95% CI: 1.048–1.566; *p* < 0.05); after adjusting for age, sex, smoking pack-year, seasonal effects and AE frequency, a 1-μg/m^3^ increase in PM_10_ was associated with a 1.200-fold increase in the adjusted OR of DH (95% CI: 0.953–1.511; *p* > 0.05). A 1-μg/m^3^ increase in PM_2.5_ was associated with a 1.287-fold increase in the crude OR of DH (95% CI: 0.979–1.693; *p* > 0.05). After adjusting for age, sex, and smoking pack-years, a 1-μg/m^3^ increase in PM_2.5_ was associated with a 1.349-fold increase in the adjusted OR of DH (95% CI: 1.008–1.806; *p* < 0.05);after adjusting for age, sex, smoking pack-year and seasonal effects, a 1-μg/m^3^ increase in PM_2.5_ was associated with a 1.356-fold increase in the crude OR of EID (95% CI: 1.005–1.827; *p* < 0.05); after adjusting for age, sex, smoking pack-year, seasonal effects and AE frequency, a 1-μg/m^3^ increase in PM_2.5_ was associated with a 1.339-fold increase in the adjusted OR of DH (95% CI: 0.946–1.896; *p* > 0.05). The logistic regression and sensitivity analysis predicting DH change in the inspiratory capacity did not show significant differences in patients with short-term exposure to air pollution (Additional file 1: Table S4a and S4b).

**Table 4:** **a** Logistic regression predicting dynamic hyperinflation change in the inspiratory capacity (△IC) of ≤ 0.100 L. **b** Sensitivity analysis predicting dynamic hyperinflation change in the inspiratory capacity (△IC) of ≤ 0.100 L

a				
Variable	Crude OR (95% CI)	*p* value	Adjusted OR (95% CI)*	*p* value
PM_10_(μg/m^3^)	1.222 (1.021–1.463)	0.028	1.269 (1.046–1.540)	0.016
PM_2.5_(μg/m^3^)	1.287 (0.979–1.693)	0.071	1.349 (1.008–1.806)	0.044
NO_2_(ppb)	1.008 (0.810–1.254)	0.945	1.011 (0.808–1.266)	0.921
O_3_(ppb)	0.709 (0.469–1.073)	0.104	0.672 (0.436–1.035)	0.071

b				
Variables	Adjust OR^#^ (95%CI)	P value	Adjust OR^¥^ (95%CI)	P value
PM_10_(μg/m^3^)	1.281 (1.048–1.566)	0.016	1.200 (0.953–1.511)	0.121
PM_2.5_(μg/m^3^)	1.356 (1.005–1.829)	0.046	1.339 (0.946–1.896)	0.100
NO_2_(ppb)	1.009 (0.803–1.269)	0.937	1.042 (0.733–1.480)	0.819
O_3_(ppb)	0.665 (0.429–1.030)	0.068	0.850 (0.464–1.556)	0.598

*PM*_*10*_ particulate matter of < 10 μm in aerodynamic diameter, *PM*_*2.5*_ particulate matter of < 2.5 μm in aerodynamic diameter; *NO*_*2*_ nitrogen dioxide; *O*_*3*_ ozone; *OR* odds ratio, *CI* confidence interval

*Multivariable logistic regression adjusted for age, sex, smoking pack-year

^**#**^Multivariable logistic regression adjusted for age, sex, smoking pack-year and seasonal effects

^**¥**^Multivariable logistic regression adjusted for age, sex, smoking pack-year, seasonal effects and AE frequency

## Discussion

This retrospective study reports three major findings: (1) 41.1% of patients with emphysema exhibited EID during the 6MWT. Patients with EID were more dyspneic and had worse lung function, severe emphysema, frequent acute exacerbation, a shorter walking distance, DH, and exposure to higher levels of air pollution than those without EID. (2) PM_10_, PM_2.5_, and NO_2_ were significantly associated with exercise-induced oxygen desaturation. (3) PM_10_ and PM_2.5_ were associated with the severity of emphysema, and associated with DH in patients with emphysema. (4) Patients who had long-term exposure with air pollutants showed significant worse scenario than the patients had short-term exposure time.

Previous research observed that O_2_ desaturation commonly occurs during the 6MWT [30], and 41.1% of patients experienced EID during the 6MWT and a shorter walking distance in our study. This was similar to previous study that reported a high prevalence of EID in patients with COPD; Jenkins et al. (2011) reported a 47% prevalence of significant oxygen desaturation in a large cohort of patients with chronic lung disease, and it was also associated with daily physical activity in patients with milder forms of COPD [31, 32].

EID in COPD is caused by multiple factors, such as severe airflow limitation, V/Q mismatch, diffusion limitation, muscle weakness, impaired daily physical activity, and DH [31, 33]. Our study found that more dyspnea sensations, worse FEV1%, severe emphysema LAA%, higher acute exacerbation, low exercise tolerance, DH, and exposure to higher levels of air pollution were significantly related to induction of O_2_ desaturation during the 6MWT. Knowledge of EID can assist clinicians in determining patients who may require O_2_ supplementation during exercise, that relieves exertional dyspnea and enhances exercise capacity. In addition to providing oxygen, we recommend avoiding exercise that is exposed to air pollution.

There is convincing epidemiological evidence that both short-term and long-term exposure to air pollutants, including PM, O_3_, carbon black, and nitrogen oxides (NO_X_), are associated with increases in respiratory morbidity and mortality [34–36]. PM_2.5_ is considered the most important pollutant, because it contains numerous toxic chemicals and penetrates deep into the lungs and cardiovascular system, posing great risks to human health. Studies reported associations of acute exacerbation, hospitalization, and mortality with acute exposure to elevated PM_2.5_ concentrations in patients with COPD [34]. Long-term exposure to ambient air pollution (PM_2.5_, NO_X_, O_3_, and carbon black) was associated with increasing emphysema as assessed by the percent with emphysema and by lung function [14]. PM_2.5_ was associated with reduced levels of and faster decline in FEV1, FVC, MMEF, and FEV1/FVC [37].

Air pollution may be associated with symptoms immediate upon exposure. It may also be associated with long-term harm affects to the body through the respiratory tract but has systemic effects[38]. Although air pollution variables appear to correlate well with the increased COPD prevalence and declining pulmonary function [39–41], less is known about the association with O_2_ desaturation during exercise in emphysematous patients. Our previous study found that air pollution (PM_2.5_, NO_2_, and O_3_) was associated with lobar emphysema, especially in the upper lobes (*p* < 0.05) [42]. PM_2.5_ can penetrate deeply into the lungs and destroy alveolar septa by generating excessive reactive oxygen species (ROS) [43]. This upper lobe-predominant distribution may include regional differences in lung physiology (ventilation/perfusion ratio, lymphatic flow, and particle clearance) [44]. The association of air pollution with the lung lobes could be related with our present similar results that 10-year exposure to PM_10_, PM_2.5_, and NO_2_ was associated with EID, but not O_3_ had a non-significant association with EID. This finding is consistent with previous studies which found that PM_10_-induced thickening of the blood-gas barrier can be explained by a reduction in the diffusion capacity of the lungs for carbon monoxide (D_L_CO) [45, 46]. It was associated with impairments of exercise capacity and oxygen saturation in COPD patients [47]. O_3_ is a reactive gas that along with other photochemical oxidants and fine particles forms a mixture termed “smog”. Ozone aggressively attacks lung tissues and is harmful to breathe [48]. In a previous 10-year prospective cohort study of children, peak O_3_ exposures were associated with reductions in FVC and FEV1 in girls with asthma and boys who spent more time outdoors [40, 49]. Sex is one of biological variable to pulmonary immune and physiologic responses after acute O_3_ challenge[50]. The longitudinal study found there were no statistically significant associations for O_3_ in asthma incidence, decline in NO_2_ and PM_2.5_ may be associated with decreased childhood asthma incidence[51]. Exposure to 0.06 ppm O_3_ causes significant decrease in mean FEV1 responses of young healthy adults[52, 53], exposure to low O_3_ concentrations could be positively associated with deleterious effects on health during physical activity. Ozone induces time losses of similar magnitude of other outdoor activities, such as walking[54]. Our results were not inconsistent with previous studies, there is no significantly correlation between O_3_ and EID, which could be that O_3_ levels in this study were lower than the United States EPA acceptable upper limit. Although O3 concentration are lower than air quality standards, there was an increase in the environmental health risk during exercise[54], despite these health risk effects, studies suggest that the health benefits of exercise be heavier than the adverse effects of pollution exposure during exercise in all but the most polluted areas[38, 55].

Next, we assessed the association between air pollution and the severity of emphysema and DH, and found that exposure to PM_2.5_ was associated with an increased emphysema severity and DH during the 6MWT. NO_2_ and PM_2.5_ were associated with the COPD prevalence in adults using GOLD criteria [13], but the mechanism responsible for the effect on COPD may differ, as NO_2_ is considered to be an airway irritant that is potentially related to the immune system and may cause respiratory tract infections and promote lung inflammation [56], Lamichhane et al.(2018) did not observe a significant association between NO_2_ levels and any pulmonary function parameter [13]. PM_2.5_ may have an unrelated effect on the airways and trigger inflammatory responses in lung tissues [57]. after adjusting for age, sex, smoking pack-year, seasonal effects and AE frequency, PM_10_ was significantly associated with an increased emphysema severity. Moreover, exposure to PM_10_ significantly increased DH during exercise. Higher daily mean PM_10_ levels were associated with an increased risk of COPD symptoms (chronic coughing, dyspnea, sputum production, wheezing, and chest tightness) [39], and lower levels of FEV1 and FVC [58]. Airflow limitation often leads to air-trapping and DH, which result in higher work of breathing due to high inspiratory threshold loads and is associated with increased oxygen consumption.

This study has some limitations. First, the number of subjects was a small simple size, and only one hospital participated in the study. Second, important factors previously demonstrated to be associated with EID in the 6MWT, such as the D_L_CO, no daily measurements or prescribed daily activities, and a lack of personal air pollution monitoring data, but we analyzed government monitoring stations close to the homes of participants. Third, previous studies have suggested that occupational exposures are important risk factors for COPD [59]. Occupation-specific data were not collected in our cross-sectional cohort study, and most our including patients were retired. Fourth, this study lacked multi-pollutant adjustment and exposure misclassification analysis, as such raising the likelihood of distorted association between exposure and documented outcome.

## Conclusions

In summary, patients with EID had more dyspnea, worse lung function, severe emphysema, frequent acute exacerbations, a shorter walking distance, dynamic hyperinflation, and exposure to higher levels of air pollution. Long-term exposure to ambient PM_10_, PM_2.5_, and NO_2_, but not O_3_, was associated with exercise-induced desaturation. PM_2.5_ was also associated with the emphysema severity, and PM_10_ and PM_2.5_ were associated with dynamic hyperinflation in patients with emphysema. Air pollution is one of the most important prevented risks to health globally. We advocate strategies for air pollution reduction and providing information to avoid exercise-induced oxygen desaturation and damage to health.

## Supplementary Information

**Additional file 1.** Additional tables.

## Data Availability

The datasets used and/or analyzed during the current study are available from the corresponding author on reasonable request.

## Abbreviations

6MWT: 6-Minute walking test
AE: Acute exacerbations
ATS: American Thoracic Society
BMI: Body-mass index
CAT: COPD assessment test
CI: Confidence interval
COPD: Chronic obstructive pulmonary disease
DH: Dynamic hyperinflation
D_L_CO: Diffusion capacity of the lungs for carbon monoxide
EID: Exercise-induced desaturation
EPA: Environmental Protection Administration
FEV1: Forced expiratory volume in the first second
FVC: Forced vital capacity
HRCT: High-resolution computer tomographic
HU: Hounsfield units
Hybrid kriging-LUR: Hybrid kriging/land-use regression
IC: Inspiratory capacity
LAA: Low attenuation area
mMRC: Modified Medical Research Council
NO: Nitrogen oxide
NO_2_: Nitrogen dioxide
O_3_: Ozone
OR: Odds ratio
PM: Particulate matter
ppm: Parts per million
ROS: Reactive oxygen species
SD: Standard deviation
SpO_2_: Standard pulse oximetry
O2: Oxygen
V/Q: Ventilation/perfusion
