# Supplementary material for: Association of air pollution exposure with exercise-induced oxygen desaturation in COPD
Source: Respir Res. 2022 Mar 31;23:77. doi: 10.1186/s12931-022-02000-1 (PMC8973558; doi:10.1186/s12931-022-02000-1)
Supplement: Supplementary file 1 — Additional file 1. Additional tables. [file 12931_2022_2000_MOESM1_ESM.doc]

**Additional file 1-R1**

| **Table S2a. Logistic regression predicting an exercise-induced desaturation of ≧4% and SpO2 of <90% during the 6-minute walking test** | | | | |
| --- | --- | --- | --- | --- |
| **variables** | **Crude OR (95%CI)** | **P value** | **Adjust OR (95%CI)** | **P value** |
| PM10(μg/m^3^) | 1.017 (0.984-1.051) | 0.323 | 1.023 (0.988-1.058) | 0.204 |
| PM2.5(μg/m^3^) | 1.021 (0.957-1.089) | 0.525 | 1.033 (0.966-1.105) | 0.346 |
| NO_2_(ppb) | 1.028 (0.936-1.129) | 0.561 | 1.038 (0.943-1.142) | 0.447 |
| O3(ppb) | 1.027 (0.973-1.085) | 0.327 | 1.037 (0.979-1.098) | 0.212 |

PM_10_, particulate matter of <10 μm in aerodynamic diameter; PM_2.5_, particulate matter of <2.5 μm in aerodynamic diameter; NO_2_, nitrogen dioxide; O_3_, ozone; OR, odds ratio; CI, confidence interval.

* Logistic regression adjusted for age, sex, smoking pack-year.

| **Table S2b. Sensitivity analysis an exercise-induced desaturation of ≧4% and SpO2 of <90% during the 6-minute walking test** | | | | |
| --- | --- | --- | --- | --- |
| **variables** | **Crude OR^#^ (95%CI)** | **P value** | **Adjust OR^¥^ (95%CI)** | **P value** |
| PM10(μg/m^3^) | 1.027 (0.990-1.065) | 0.149 | 1.023 (0.985-1.063) | 0.242 |
| PM2.5(μg/m^3^) | 1.036 (0.967-1.110) | 0.312 | 1.029 (0.956-1.108) | 0.444 |
| NO_2_(ppb) | 1.036 (0.940-1.141) | 0.477 | 1.018 (0.917-1.131) | 0.732 |
| O3(ppb) | 1.046 (0.985-1.111) | 0.141 | 1.045 (0.981-1.114) | 0.170 |

**^#^**multivariable logistic regression adjusted for age, sex, smoking pack-year and seasonal effects.

**^¥^**multivariable logistic regression adjusted for age, sex, smoking pack-year, seasonal effects and AE frequency.

| **Table S3a. Logistic regression predicting severe emphysema low attenuation area (LAA) of ≧20%** | | | | |
| --- | --- | --- | --- | --- |
| **variables** | **Crude OR (95%CI)** | **P value** | **Adjust OR (95%CI)** | **P value** |
| PM10(μg/m^3^) | 1.011 (0.976-1.046) | 0.556 | 1.011 (0.975-1.048) | 0.559 |
| PM2.5(μg/m^3^) | 1.025 (0.958-1.098) | 0.472 | 1.025 (0.955-1.101) | 0.488 |
| NO_2_(ppb) | 1.028 (0.931-1.135) | 0.584 | 1.042 (0.938-1.157) | 0.441 |
| O3(ppb) | 1.006 (0.950-1.065) | 0.850 | 0.992 (0.934-1.053) | 0.786 |

PM_10_, particulate matter of <10 μm in aerodynamic diameter; PM_2.5_, particulate matter of <2.5 μm in aerodynamic diameter; NO_2_, nitrogen dioxide; O_3_, ozone; OR, odds ratio; CI, confidence interval.

* Logistic regression adjusted for age, sex, smoking pack-year

| **Table S3b. Sensitivity analysis predicting severe emphysema low attenuation area (LAA) of ≧20%** | | | | |
| --- | --- | --- | --- | --- |
| **variables** | **Adjust OR^#^ (95%CI)** | **P value** | **Adjust OR^¥^ (95%CI)** | **P value** |
| O3(ppb) | 1.019 (0.984-1.059) | 0.324 | 1.015 (0.975-1.057) | 0.474 |
| PM2.5(μg/m^3^) | 1.036 (0.963-1.114) | 0.347 | 1.029 (0.952-1.113) | 0.472 |
| PM10(μg/m^3^) | 1.049 (0.943-1.167) | 0.363 | 1.043 (0.931-1.169) | 0.468 |
| NO_2_(ppb) | 1.003 (0.942-1.068) | 0.926 | 1.002 (0.939-1.069) | 0.952 |

**^#^**multivariable logistic regression adjusted for age, sex, smoking pack-year and seasonal effects.

**^¥^**multivariable logistic regression adjusted for age, sex, smoking pack-year, seasonal effects and AE frequency.

| **Table S4a. Logistic regression predicting dynamic hyperinflation change in the inspiratory capacity (△IC) of ≦0.100 L** | | | | |
| --- | --- | --- | --- | --- |
| **variables** | **Crude OR (95%CI)** | **P value** | **Adjust OR (95%CI)** | **P value** |
| PM10(μg/m^3^) | 1.027 (0.988-1.067) | 0.177 | 1.025 (0.984-1.068) | 0.238 |
| PM2.5(μg/m^3^) | 1.031 (0.951-1.118) | 0.456 | 1.025 (0.942-1.115) | 0.567 |
| NO_2_(ppb) | 0.981 (0.885-1.087) | 0.981 | 0.982 (0.884-1.092) | 0.739 |
| O3(ppb) | 1.046 (0.983-1.113) | 0.154 | 1.031 (0.965-1.101) | 0.366 |

PM_10_, particulate matter of <10 μm in aerodynamic diameter; PM_2.5_, particulate matter of <2.5 μm in aerodynamic diameter; NO_2_, nitrogen dioxide; O_3_, ozone; OR, odds ratio; CI, confidence interval.

* Logistic regression adjusted for age, sex, smoking pack-year.

| **Table S4b. Sensitivity analysis predicting dynamic hyperinflation change in the inspiratory capacity (△IC) of ≦0.100 L** | | | | |
| --- | --- | --- | --- | --- |
| **variables** | **Adjust OR^#^ (95%CI)** | **P value** | **Adjust OR^¥^ (95%CI)** | **P value** |
| O3(ppb) | 1.027 (0.985-1.074) | 0.216 | 1.038 (0.991-1.087) | 0.111 |
| PM2.5(μg/m^3^) | 1.026 (0.942-1.118) | 0.557 | 1.039 (0.943-1.146) | 0.437 |
| PM10(μg/m^3^) | 0.988 (0.886-1.101) | 0.826 | 0.965 (0.852-1.093) | 0.578 |
| NO_2_(ppb) | 1.025 (0.959-1.096) | 0.460 | 1.034 (0.962-1.112) | 0.357 |

**^#^**multivariable logistic regression adjusted for age, sex, smoking pack-year and seasonal effects.

**^¥^**multivariable logistic regression adjusted for age, sex, smoking pack-year, seasonal effects and AE frequency.
